# *Saccharomyces cerevisiae* Genetics Predicts Candidate Therapeutic Genetic Interactions at the Mammalian Replication Fork

**DOI:** 10.1534/g3.112.004754

**Published:** 2013-02-01

**Authors:** Derek M. van Pel, Peter C. Stirling, Sean W. Minaker, Payal Sipahimalani, Philip Hieter

**Affiliations:** Michael Smith Laboratories, University of British Columbia, Vancouver, British Columbia, Canada, V6T1Z4

**Keywords:** synthetic lethality, DNA replication, cancer therapy, yeast genomics, chromosome instability

## Abstract

The concept of synthetic lethality has gained popularity as a rational guide for predicting chemotherapeutic targets based on negative genetic interactions between tumor-specific somatic mutations and a second-site target gene. One hallmark of most cancers that can be exploited by chemotherapies is chromosome instability (CIN). Because chromosome replication, maintenance, and segregation represent conserved and cell-essential processes, they can be modeled effectively in simpler eukaryotes such as *Saccharomyces cerevisiae*. Here we analyze and extend genetic networks of CIN cancer gene orthologs in yeast, focusing on essential genes. This identifies hub genes and processes that are candidate targets for synthetic lethal killing of cancer cells with defined somatic mutations. One hub process in these networks is DNA replication. A nonessential, fork-associated scaffold, *CTF4*, is among the most highly connected genes. As Ctf4 lacks enzymatic activity, potentially limiting its development as a therapeutic target, we exploited its function as a physical interaction hub to rationally predict synthetic lethal interactions between essential Ctf4-binding proteins and CIN cancer gene orthologs. We then validated a subset of predicted genetic interactions in a human colorectal cancer cell line, showing that siRNA-mediated knockdown of *MRE11A* sensitizes cells to depletion of various replication fork-associated proteins. Overall, this work describes methods to identify, predict, and validate in cancer cells candidate therapeutic targets for tumors with known somatic mutations in CIN genes using data from yeast. We affirm not only replication stress but also the targeting of DNA replication fork proteins themselves as potential targets for anticancer therapeutic development.

Synthetic lethality describes a genetic interaction between two independently viable mutations whose combination results in lethality. Synthetic lethal (SL) relationships usually indicate a shared biological function, and they have been used as a research tool of geneticists for decades. In the past 15 years, the concept of synthetic lethality as a therapeutic strategy has gained popularity, especially in the rational targeting of cancers with known somatic mutations (Brough *et al.* 2011; Hartwell *et al.* 1997; Kaelin 2005). Cancerous cells carry mutations that differentiate them from surrounding normal cells. Thus, devising a strategy based on SL interactions is a rational approach to selectively target cancer cells. In this scenario, validated SL partners of a cancer-mutated gene are targeted to selectively kill tumor cells while, ideally, leaving neighboring normal tissues relatively unaffected. Although it is likely that current chemotherapies inadvertently exploit genotypic changes to exert their antiproliferative effects, there are relatively few examples of strictly SL-based therapies in clinical trials (Brough *et al.* 2011; Kaelin 2005).

Along with oncogenes and tumor-suppressors, chromosome instability (CIN) genes are a third class of cancer mutations that promote oncogenesis by destabilizing the genome. CIN will increase the mutational space explored by dividing pretumor cells and thereby increase the likelihood that the mutations required for malignancy will occur and be selected from the mutant population (Loeb 2011; Stirling *et al.* 2012a; Stratton *et al.* 2009). CIN is an attractive target for chemotherapies because CIN mutations represent a sublethal hit on the essential process of genome replication and segregation and thus could conceivably be enhanced to lethality by therapeutics. Moreover, aneuploidy is seen in >90% of solid tumors and in the majority of leukemias, suggesting that therapies specifically targeting CIN could have a broad spectrum of action (Weaver and Cleveland 2006; Gordon *et al.* 2012). Genes that maintain chromosome stability are found in all cells and are highly conserved among eukaryotes, probably due to the essential nature of DNA replication, repair, and segregation. Therefore, the yeast *Saccharomyces cerevisiae* is an excellent model in which to study CIN specifically, whereas cancer-associated cellular pathways specific to multicellular organisms (*e.g.*, growth factor signaling, apoptosis) must be modeled in more complex systems.

*S. cerevisiae* has been a proving ground for genomic technologies. Two transformative events in yeast genetics have been the development of the deletion mutant collection and, subsequently, synthetic genetic array (SGA). These technologies together enable systematic assessment of genetic interactions in a genome-wide pairwise fashion (Tong *et al.* 2001, 2004) and high-throughput screening of numerous compounds against haploid or homozygous nonessential gene mutants and heterozygous diploid essential gene mutants, linking chemical sensitivities to specific genetic backgrounds (Giaever *et al.* 2002, 2004; Hillenmeyer *et al.* 2008; Parsons *et al.* 2006; Smith *et al.* 2009). Before the advent of these technologies, Hartwell and colleagues suggested the use of yeast to profile the genetic determinants of cellular sensitivity to chemotherapeutic compounds and the potential for synthetic genetic interactions to predict therapeutic targets (Hartwell *et al.* 1997). Since describing SGA technology, a large percentage of possible pair-wise genetic interactions in yeast have been tested by high-throughput SGA screens (Costanzo *et al.* 2010). Naturally, this analysis encompasses screens of many yeast orthologs of human cancer genes, and as such predicts many second-site SL partner genes that could, in principle, be therapeutic targets.

Prediction of SL interactions *a priori* also has been successful in identifying therapeutic targets, as exemplified by the identification of poly-ADP-ribose polymerase (PARP) as a therapeutic target for cancers with *BRCA1* or *BRCA2* mutations (Bryant *et al.* 2005; Farmer *et al.* 2005). The example of PARP underscores the importance of elucidating genetic interaction hubs and analyzing genetic networks to define new therapeutic opportunities and targets. The creation of SL networks has the potential to identify new therapeutic targets, explain the genetic basis of existing therapies, and aid the understanding of associations of particular mutations with prognosis. To identify candidate therapeutic genetic interactions, a popular screening approach has been to use whole-genome shRNA libraries on paired human cell lines differing only at a single causative mutant locus (*e.g.*, KRAS-transformed cell lines) (Luo *et al.* 2009; Scholl *et al.* 2009), although this approach is limited by the availability of paired cell lines and the cost of the screens.

To identify common weaknesses of CIN gene mutations and predict novel candidate therapeutic processes and target genes from yeast data, we developed chemical and genetic interaction maps derived from high-throughput genetic screens conducted in this study and from the literature. Overall, two broad and connected processes dominate the CIN genetic interaction network: DNA replication/repair and the mitotic machinery. Consistently, we identify new hub genes that also fall into these two categories. Focusing on the DNA replication fork, we show that mutations in essential physical interaction partners of a hub gene, *CTF4*, recapitulate cancer-relevant *ctf4*Δ negative genetic interactions. We confirm several of these interactions in a human colorectal cancer cell line depleted for the cancer gene *MRE11A*. Although a complete genetic interaction map should elucidate the best SL targets for cancer gene orthologs, we show that our existing knowledge of genetic networks suggest novel candidate therapeutic targets that can be confirmed by directed experiments in mammalian cells.

## Materials and Methods

### Yeast strains and growth

Yeast strains and plasmids are listed in Supporting Information, Table S1. Yeast were grown on rich media (YPD) with the appropriate drug selection or on synthetic complete media lacking the appropriate amino acids for selection. For temperature-sensitive strains, all manipulations were performed at 25° except where indicated to measure growth phenotypes. For spot assays, 10-fold serial dilutions of the indicated strains were spotted on rich media with or without addition of a DNA damaging chemical.

### SGA and chemical sensitivities

SGA was performed essentially as described (Tong *et al.* 2004). For chemical screening, arrays of yeast mutants were pinned in triplicate onto YPD containing either 0.01% methylmethane sulfonate (MMS), 50 mM hydroxyurea (HU), 10 μg/mL benomyl, or 1 ng/mL rapamycin. After 24 hr growth, each plate was again pinned onto chemical-containing media in triplicate leading to nine total replicates passaged on YPD + chemical. Plate images were collected after another 24 hr growth on a flatbed scanner. Image analysis and scoring for both SGA and chemical sensitivities was performed as described (McLellan *et al.* 2012; Stirling *et al.* 2011). Chemicals were selected based on diverse mechanisms representing genotoxic or nongenotoxic anticancer strategies (Table 1).

**Table 1:** Rationale for chemicals used in genome-wide analysis

Chemical	Mode of Action	Analogues in Chemotherapy
Methyl methanesulfonate	Alkylating agent; directly damages DNA bases	Nitrogen-mustard based (*e.g.*, ifosfamide, chlorambucil); other (*e.g.*, temozolomide)
Hydroxyurea	Inhibitor of ribonucleotide reductase; causes stalled replication forks due to reduced nucleotide pool	Hydroxyurea (*i.e.*, marketed as Droxia, Hydrea)
Benomyl	Binds tubulin heterodimers preventing microtubule assembly	Vinblastine, vincristine, vinorelbine, vindesine, paclitaxel
Rapamycin	Binds FK506-binding protein to inhibit TORC1 signaling	Sirolimus and derivatives [*e.g.*, everolimus (Afinitor); temsirolimus (Torisel)]

### Genetic network analysis

Interaction networks were generated using Cytoscape (Shannon *et al.* 2003). Genetic interaction data for CIN genes was extracted from the DryGIN database or our own SGA screen results (Costanzo *et al.* 2010; Koh *et al.* 2010).

### Fluorescence microscopy

Live, logarithmically growing cells were mounted on concanavalin A−coated slides and imaged using the YFP filter set as described (Carroll *et al.* 2009; Stirling *et al.* 2012b). Images were collected in Metamorph (Molecular Devices) and analyzed in ImageJ (http://rsbweb.nih.gov/ij/).

### Cell culture and siRNA transfections

HCT116 cells (ATCC) were grown in McCoy’s 5A medium supplemented with 10% fetal bovine serum. ON-TARGET*plus* siRNA pools (Dharmacon) were transfected using DharmaFECT I (Dharmacon) at 25 nM, such that the total siRNA concentration was always 50 nM (for single transfections, nontargeting siRNA was used to supplement). Culture medium was replenished approximately 8 hr posttransfection, and cells were transferred to 96-well optical bottom plates approximately 24 hr posttransfection. When appropriate, 10 μM mirin (Sigma-Aldrich) was added to cells 24 hr after replating.

### Genetic interaction determination

Cells in optical-bottom plates were fixed 72 hr postseeding in 4% paraformaldehyde/phosphate-buffered saline, and nuclei were labeled with Hoechst 33342 at 500 ng/mL in phosphate-buffered saline. Stained nuclei were counted using a Cellomics Arrayscan VTI fluorescence imager as described previously (McManus *et al.* 2009). To determine the presence of a SL interaction, the proliferative defect was calculated, and is defined as

$$\text{1}-\frac{\mathrm{Proliferation predicted by a multiplicative model}}{\mathrm{Observed proliferation}}$$

where the predicted proliferation was the product of the proliferation of the two individual gene knockdowns, following a multiplicative model of genetic interactions (Baryshnikova *et al.* 2010). SL interactions were scored as a proliferative defect of 15% or greater than the predicted value. For colony formation assays, cells were fixed in 0.01% w/v crystal violet/95% ethanol approximately 10 d after plating.

## Results

### Selective killing of CIN mutants by genome-destabilizing chemicals

Recent work identifying and evaluating gene−drug interactions in cancer has revealed that compounds targeting a specific genotype typically yield better selective killing than drugs having a more general cytotoxic effect (Barretina *et al.* 2012; Garnett *et al.* 2012). Given the prevalence of CIN in cancer, identification of a specific second-site target whose inhibition causes synthetic lethality in cancers with a broad spectrum of CIN mutations would be ideal. Current chemotherapeutic strategies often exploit genotoxic compounds, and it seems reasonable that these compounds are selective because of underlying CIN mutations and/or the aneuploidy status of tumor cells. To confirm that CIN genetic backgrounds are indeed sensitive to genotoxins, we generated a comprehensive profile of the genotypes targeted by cytotoxic therapeutic strategies whose effects challenge genome integrity pathways: specifically, DNA replication, repair, and mitosis. We focused on four distinct classes of chemical: MMS, HU, benomyl, and, as a nongenotoxic control, rapamycin (Table 1). Because sensitivity data are already available for all nonessential gene deletions, we tested only 1945 DAmP (*i.e.*, Decreased Abundance by mRNA Perturbation) and ts (temperature-sensitive) alleles in essential genes, representing ~90% of all essential yeast genes (Table 1); (Ben-Aroya *et al.* 2008; Breslow *et al.* 2008; Li *et al.* 2011). Adding these data for essential genes is particularly relevant to CIN because nearly half of reported CIN genes are essential (Stirling *et al.* 2011).

**Figure 1:**
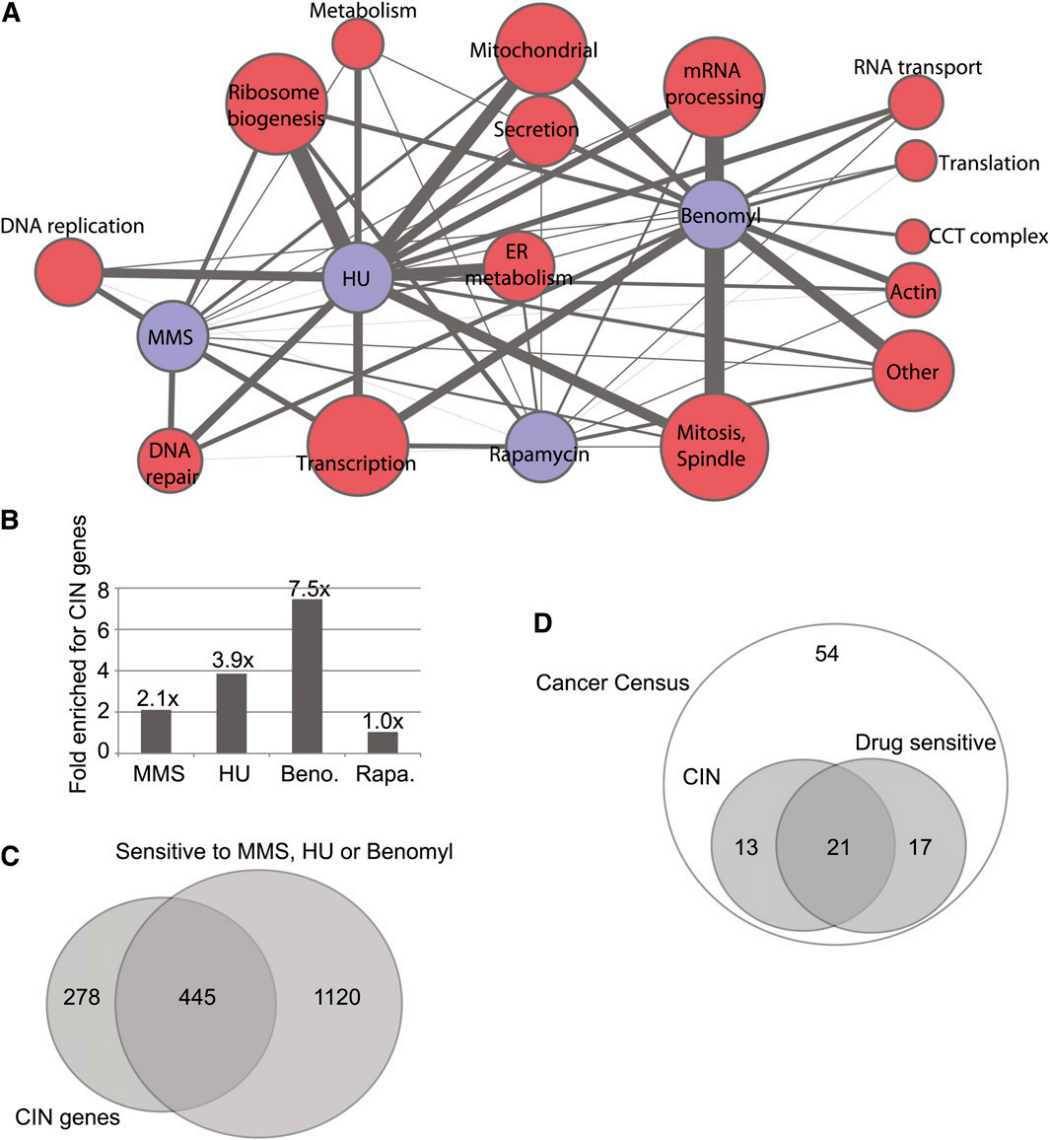
Sensitivities of yeast mutants to genotoxic chemicals. (A) Gene ontology−derived cellular functional groups uncovered by chemical screening of essential gene mutants. The network represents the raw data in supporting Table S2. Chemicals are indicated with blue nodes. Red nodes represent functional groups where node size indicates the number of genes in that group and edge thickness indicates the number of connections to a particular chemical. (B) Compilation of new chemical sensitive genes with the literature highlights the enrichment of CIN genes within genotoxic drug sensitivity profiles. Enrichment indicates the quotient of the percentage of CIN genes in each chemical sensitivity list (Table S3) and the percentage of CIN genes in the entire genome. (C) Overlap of MMS, HU, or benomyl-sensitive mutants with known CIN genes. (D) Number of CIN genes with orthologs on the cancer gene census that are sensitive to one of the chemicals tested.

Growth of the essential gene mutant strains in the presence of chemicals was measured in high-throughput array format (see *Materials and Methods*). A total of 123, 122, 47, and 33 genes met our stringent cut-off for a negative chemical−genetic interaction with HU, benomyl, MMS, and rapamycin, respectively (Table S2). Grouping the essential genes sensitized to each chemical by gene ontology allowed us to build a network of pathways affected by the four chemicals (Figure 1A). Many expected interactions emerged: for example, MMS and HU impacted DNA replication and repair, whereas benomyl was strongly associated with mitotic spindle defects (Figure 1A). However, analysis of the essential genes also provided several new insights: for example, RNA processing mutants were highly sensitized to benomyl. This is potentially due to the presence of an intron in the *TUB1* gene that, if improperly spliced, would lead to a toxic excess of β tubulin relative to α tubulin (Biggins *et al.* 2001; Burns *et al.* 2002).

These observations are restricted to our analysis of essential genes; to gain a global view, we pooled our new data with the literature compiled in the *Saccharomyces* Genome Database (www.yeastgenome.org). This created lists of 519, 750, 296, and 772 genes sensitive to HU, MMS, benomyl, and rapamycin, respectively (Table S3). When we enumerated genes that are sensitive to one of the four test chemicals and also have a CIN phenotype, we saw clear enrichment of CIN genes sensitive to benomyl, MMS, and HU but no enrichment of CIN genes impacted by the nongenotoxic control rapamycin (Figure 1B). Indeed, benomyl-sensitive mutants exhibit a greater than sevenfold enrichment of CIN genes. This may be explained by the fact that benomyl is most likely to cause whole chromosome loss, as it functions to disrupt microtubules, and chromosome loss is a common endpoint measure in CIN assays (Stirling *et al.* 2011; Yuen *et al.* 2007). The majority of all reported CIN genes (*i.e.*, 445/692; 64%) were sensitized to at least one of the genome destabilizing drugs (Figure 1C). Moreover, like yeast CIN mutations, essential genes were enriched among the mutations with sensitivity to MMS, HU, and benomyl but not rapamycin (Stirling *et al.* 2011). Specifically focusing on the yeast orthologs of cancer gene census genes, that is, genes believed to play a causative role in tumorigenesis (Futreal *et al.* 2004), we also observed that the majority of CIN genes in this subset were sensitive to one of the genome destabilizing chemicals (Figure 1D). Although these findings are unsurprising, given the known modes of action of MMS, HU, and benomyl, they support the concept that chemotherapeutic strategies that target genome stability can be broadly effective and will take advantage of the specific genetic background of the tumor to yield selective killing.

### Cancer−gene ortholog-centered analysis of the SL network

**Figure 2:**
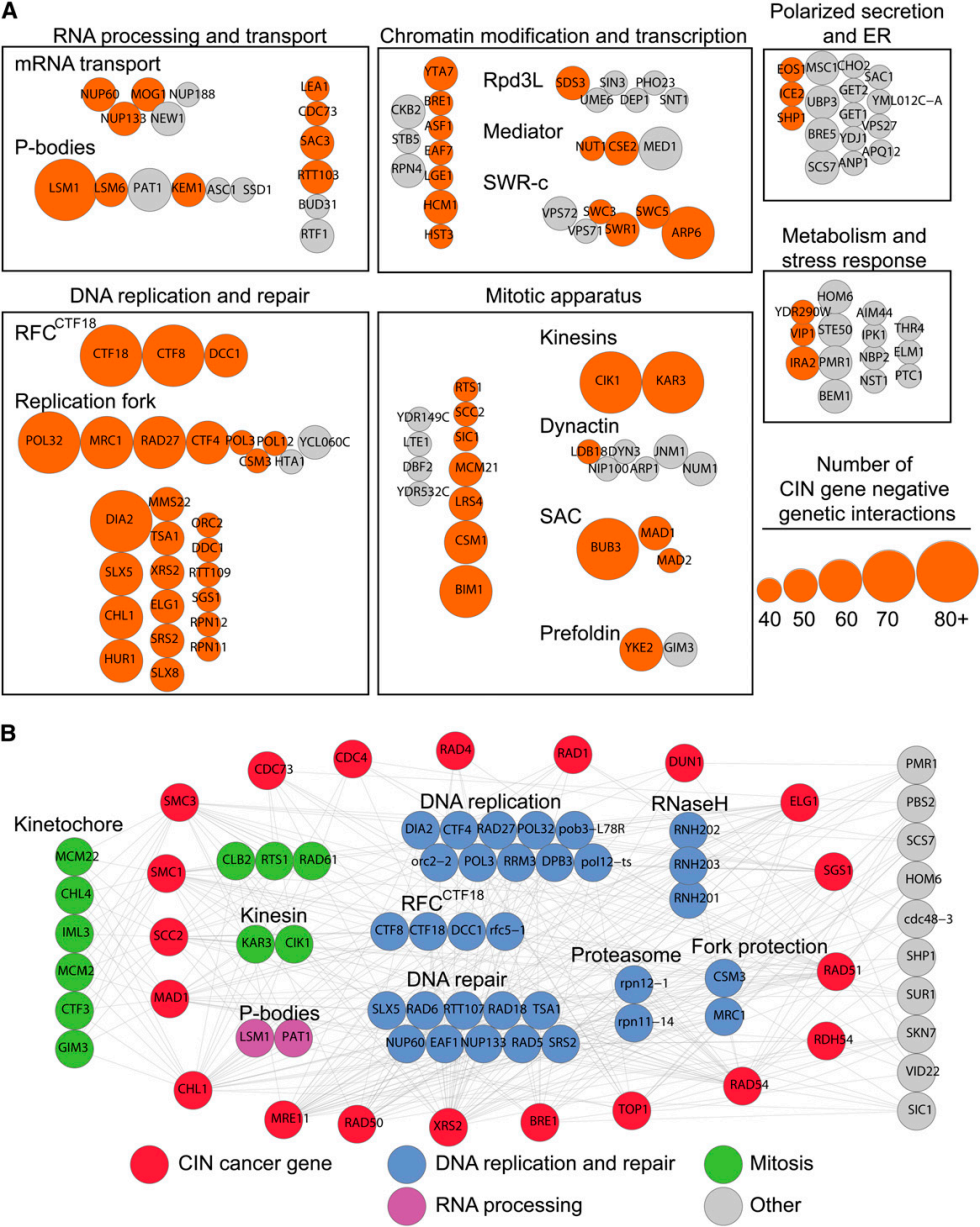
The network of negative genetic interactions with CIN genes. (A) Using publicly-available data, we grouped genes connected to 40 or more CIN genes by negative genetic interactions into functional categories primarily on the basis of Gene ontology biological processes. Node size denotes the relative number of interactions between a hit and the 461 CIN genes (legend on the right). Orange nodes, CIN genes; gray nodes, non-CIN genes. (B) Genes with five or more negative genetic interactions with a selection of CIN cancer-gene orthologs were grouped according to cellular functions/protein complexes. Node color key is indicated at the bottom and functional subgroups are labeled.

Because broad-spectrum genotoxins selectively kill cells with CIN genetic backgrounds (Figure 1), SL interactions should be valuable tools to predict additional, and potentially more specific, therapeutic agents for targeting cells with CIN. Any gene that is SL with a CIN gene is thus a possible second-site target for anticancer therapeutic development (Hartwell *et al.* 1997). Ideally, such targets would also be broad spectrum, that is, the second-site gene would be SL with many cancer CIN genes. To begin to identify such highly-connected SL partner genes in yeast, we extracted the genetic interactions published specifically for CIN genes from the DryGIN database (Costanzo *et al.* 2010; Koh *et al.* 2010). We selected negative genetic interactions with the 461 CIN genes represented in the Costanzo dataset, then set an arbitrary filter of ≥40 negative interactions with CIN genes to highlight the most connected genes (Figure 2A). This approach yielded a simple network with four large and two smaller groups. Not surprisingly, the two cellular processes that dominate the CIN-SL landscape are ‘DNA replication and repair’ and the ‘mitotic apparatus.’ ‘Chromatin modification and transcription’ and ‘RNA processing and transport’ are the two other large groups of genes. Two smaller groups of highly connected genes were classified as relating to ‘polarized secretion and ER’ and ‘metabolism and stress response’ (Figure 2A). The majority of strongly interacting genes were themselves CIN genes and DNA replication and mitosis were the groups most highly connected to CIN (Figure 2A). Within the broad groups there exist several genetic hubs representing protein complexes or biological structures, such as mitotic kinesins, prefoldin, the Ctf18 replication factor C (RFC^Ctf18^) complex, and the DNA replication fork. Several surprising or previously unappreciated hubs also emerged, including the cytoplasmic Processing body (P-body) that degrades mRNAs in response to stress. The *LSM1* gene was among the most highly connected to CIN and has recently been implicated in the response to DNA replication stress both through cytological studies and via its role in degrading histone mRNAs (Herrero and Moreno. 2011; Tkach *et al.* 2012).

Hubs identified using the approach described previously represent potential targets for anticancer therapeutic development, as they are SL with many yeast CIN genes; however, not all human orthologs of yeast CIN genes are mutated in cancer. Thus, to confirm that the identified CIN-SL hubs could in principle selectively target CIN cancer mutations, we reanalyzed the data using a smaller network of known CIN cancer−gene orthologs (Figure 2B). We selected 20 yeast orthologs of CIN genes that are recurrently mutated in various cancers (*e.g.*, *CDC4*, *MRE11*, cohesins) or are represented in the cancer gene census (Futreal *et al.* 2004). Filtering for highly connected SL targets of these cancer genes highlights DNA replication and repair genes, including the replication fork protection complex, the replication initiation complex, the RFC^Ctf18^, the RNaseH complex, and two proteasome subunits (Figure 2B). Genes with kinetochore and other mitotic functions also emerge, although they are primarily connected to CIN cancer genes with cohesion and spindle functions. Remarkably, the P-body components *LSM1* and *PAT1* remain highly connected to this cancer-specific network, raising the possibility that P-body disruption could have therapeutic value through selective killing of CIN cancers. The highly connected second-site genes identified by focusing on orthologs of genes known to be mutated in cancer, as opposed to the unbiased approach described previously, may represent more high-priority targets.

To ascertain whether these general observations might extend to newly described essential CIN cancer genes, we carried out SGA analysis on *taf1-1*. The essential gene *TAF1* encodes the largest subunit of TFIID, and the *taf1-1* mutation causes increased CIN (Stirling *et al.* 2011). The human genome encodes two closely related *TAF1* orthologs, *TAF1* and *TAF1L*, which together are mutated in >10% of colon adenocarcinoma and >20% of lung squamous cell carcinoma [TCGA data via MSKCC www.cbioportal.org/public_portal (The Cancer Genome Atlas Research Network 2012)]. Although the significance of the TAF1/1L mutations is not known, the frequency of mutations suggest that TAF1/1L may play some functional role in modulating the phenotype of cancer cells. Regardless, the mutations in TAF1/1L might represent an Achilles’ heel for SL targeting of a CIN gene mutated in many tumors. SGA analysis of *taf1-1* revealed that, in addition to the expected interactions with transcription initiation and chromatin remodeling (*e.g.*, TFIID, mediator, SWR-c), *taf1-1* also exhibited negative genetic interactions with spindle checkpoint (*BUB3*), DNA replication and P-body genes (*LSM1*; Figure S1). This pattern is broadly similar to that seen for many other, unrelated CIN cancer genes in Figure 2B. Overall, our network analysis, supported by the example of *TAF1*, suggests that common features underlie the genetic interaction spectrum of mutants with genome instability. Specific inhibition of genes functioning within these ‘hub’ processes could therefore be broadly useful as therapy.

### Expansion of SL network reveals new targets in familiar pathways

Other than *TAF1*, the genetic networks described previously were generated using publicly-available data, which until recently has been comprised almost exclusively of screens with nonessential gene deletion mutations. The relative paucity of essential gene mutants in the published SL network thus represents an opportunity to identify new highly connected second-site candidate therapeutic targets. We sought to expand the known genetic interaction space for important cancer gene orthologs by direct screening for interactions with essential genes. We screened nine query mutations whose human orthologs are mutated in cancer (*mad1*Δ, *bub1*Δ, *chl1*Δ, *rad51*Δ, *cdc73*Δ, *sgs1*Δ, *elg1*Δ, *mre11*Δ, and *smc3-42*) against a miniarray of 1161 DAmP or ts-alleles in 923 essential yeast genes (Breslow *et al.* 2008; Li *et al.* 2011).

**Figure 3:**
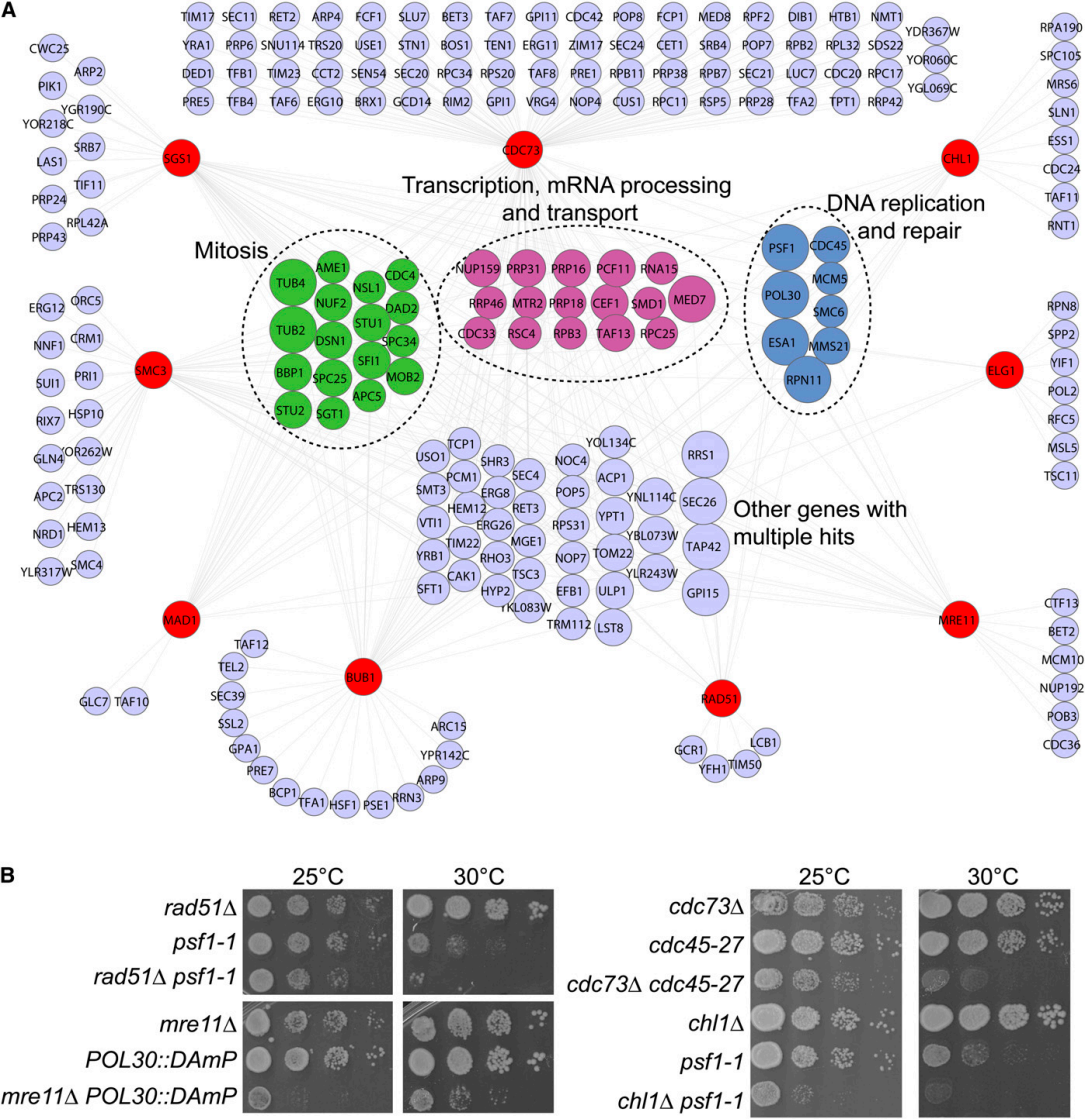
Essential gene genetic interactions with nine CIN cancer gene orthologs. (A) Network of essential gene negative interactions with nine indicated query mutations in CIN cancer-gene orthologs (red nodes). Those nodes with connections to two or more queries are grouped in the center according to functional similarities, color-coded as in Figure 2A. Node size increases with the number of connections to the nine query mutants. (B) Validation of selected genetic interactions by spot dilution assays. Double mutants were isolated at the permissive temperature of 25° and spotted at the indicated temperatures to reveal interactions.

SGA analysis of the essential mutant array retrieved common interacting partners of the nine query genes functioning in transcription, DNA replication, and mitosis after filtering for reproducible and strong interactions (*P* < 0.05, Experimental-Control: −0.20; Figure 3A and Table S4). This finding indicates that screening essential genes is likely to expand the suite of candidate targets present in the existing biological pathways outlined in Figure 2. The functional group with, on average, the most highly connected constituents (*i.e.*, large nodes in Figure 3A) was DNA replication and repair. Because the hubs in this network in principle represent candidate therapeutic targets that could selectively target tumor cells with diverse CIN mutations, we chose to focus on DNA replication and repair genes. We validated selected interactions by tetrad analysis and spot dilution assays at a semipermissive temperature (30°; examples in Figure 3B and summarized in Table S5). Our results confirm members of the DNA replication fork as hubs for genetic interactions with many cancer gene orthologs and suggest other potential hub pathways.

### Prediction of SL interactions at the DNA replication fork

In previous analyses authors also have recognized the importance of DNA replication fork proteins in genetic networks of cancer genes (Yuen *et al.* 2007). One of the most highly connected replication fork proteins is Ctf4, which appears to function as a scaffold during DNA replication and repair, acting as a hub of protein−protein interactions (Im *et al.* 2009; Mimura *et al.* 2010; Zhu *et al.* 2007). To attempt to separate these functions of Ctf4, we sequenced and characterized a set of nine *CTF4* mutant alleles that are represented by four missense mutants and five nonsense mutants, isolated in the original CTF screen (Figure S2) (Spencer *et al.* 1990). This analysis primarily revealed that the ability to bind its partner proteins is crucial for Ctf4 to perform its cellular genome integrity function (Figure S2, Table S6, and File S1). SGA analysis of *ctf4*Δ against an array of essential gene mutants confirmed the enrichment of cellular genome stability pathways, including those whose orthologs are recurrently mutated in tumors (*e.g.*, cohesins, *CDC4*, *MRE11*; Figure S3, Table S5, Table S7, and Table S8).

In a recent, related study we found that *CTF4* genetic interactions with the CIN cancer genes *MRE11A*, *CDC4*, and *BLM* are conserved in human cells (van Pel *et al.* 2013). However, given the lack of a quantifiable biochemical activity for Ctf4/WDHD1, it is not clear how biochemical inhibitor screening would be performed. Attempts to develop cell-based screens using *S. cerevisiae* restoration-of-growth (Balgi and Roberge 2009) have found that expression of human WDHD1 is not toxic to yeast (Figure S4). Thus, inhibition of Ctf4/WDHD1 itself will require significant further experimentation and alternative approaches will need to be developed.

Members of the same complex have been shown to share genetic interactions (Collins *et al.* 2007; Tong *et al.* 2004); therefore, understanding the biological context of Ctf4 function and its genetic interaction network enables prediction of new SL interactions with its physical interaction partners. In principle, validating SL interactions between cancer CIN gene orthologs and functional partners of Ctf4 could identify better therapeutic targets than Ctf4 itself, such as those having enzymatic activity. Pathway-based SL prediction using functional data from yeast presents a means to circumvent the issue of being unable to screen for small-molecule inhibitors of highly-connected and promising targets such as Ctf4.

**Figure 4:**
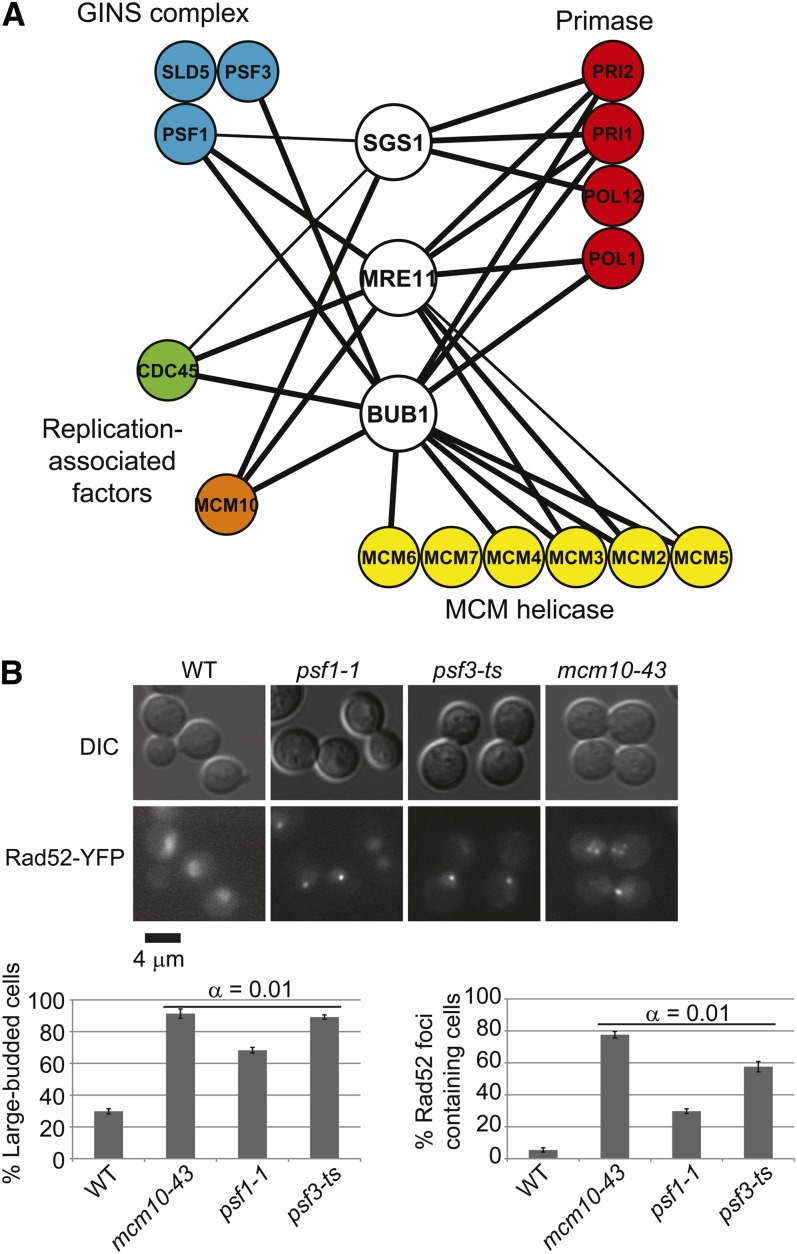
Functional neighbors predict surrogate genetic interactions. (A) Physical interactions predict genetic interactions. Indicated heterozygous diploid mutants were subjected to random spore analysis. Thick edges, double mutants are inviable at a semipermissive temperature. Thin edges, double-mutant colonies are smaller at a semipermissive temperature. (B) Rad52 foci and G2/M cell-cycle arrest in GINS and *MCM10* mutants. Top, representative DIC and YFP images of the indicated strains. Foci are evident as bright puncta in the mutant panels. Bottom, quantification of G2/M arrest large-budded cells (left) and cells with Rad52-YFP foci (right). α indicates the result of Tukey post-hoc analysis of a one-way analysis of variance. Each mutant was significantly different than the WT.

Ctf4 physically interacts with the MCM complex, the repair factor Mms22, the POLα primase complex, the GINS complex, and the replication-associated factors Cdc45 and Mcm10 (Im *et al.* 2009; Mimura *et al.* 2010; Zhu *et al.* 2007). Our approach predicts that mutants in these Ctf4-interacting proteins would mimic some of the genetic interactions of *ctf4*Δ with cancer-gene orthologs. Figure 4A summarizes the results of direct testing for negative genetic interactions between the cancer-gene orthologs *mre11*Δ, *bub1*Δ, and *sgs1*Δ and mutant alleles of *CDC45*, *MCM10*, primase, MCM, or GINS complexes. In almost every case, interactions are observed between each cancer−gene ortholog and at least one member of the Ctf4-interacting complexes. Because all of the Ctf4-interacting genes are essential, partial loss-of-function (*i.e.*, DAmP or ts) alleles are used, potentially explaining why not all genes tested show interactions. Regardless, the result strongly validates the approach of targeting pathways and functional partners of hub genes for SL killing of cancer cells when the original candidate target gene is not immediately amenable to small-molecule inhibitor development.

In considering the potential mechanism of lethality, we noted that mutations of *CDC45* and subunits of primase or Mcm2-7 have previously been associated with G2/M cell cycle arrest and increased Rad52 foci (Stirling *et al.* 2012b). Direct testing of ts alleles of *MCM10* and the GINS subunits *PSF1* and *PSF3* show that these mutants also cause dramatic increases in Rad52 foci and a G2/M cell cycle arrest (Figure 4B). Therefore, all of the Ctf4 partners tested have a common requirement for increased recombinational DNA repair and a functioning G2/M cell cycle checkpoint.

### MRE11A inhibition or depletion sensitizes colorectal cancer cells to perturbation of replisome genes

**Figure 5:**
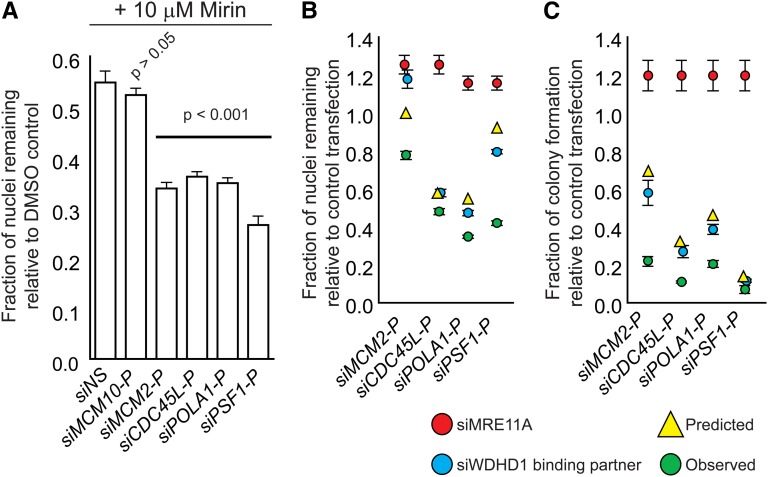
Yeast genetic interactions predicted by physical interactions are evolutionarily conserved. (A) Chemical inhibition of MRE11 by mirin sensitizes cells to depletion of Ctf4/WDHD1 physical interactors. Data were analyzed by one-way analysis of variance followed by a Tukey test. *P* values indicate differences between test and control transfections. NS, nonsilencing control siRNA. (B and C) siRNA-mediated knockdown of MRE11A sensitizes cells to depletion of Ctf4/WDHD1 physical interactors. Experiments were carried out as in (A), except that MRE11A was depleted by siRNA. Red circles, results of MRE11A transfection. Blue circles, results of transfection with siRNA targeting gene indicated along the *x*-axis. Yellow triangles, predicted growth of double siRNA-treated cells, assuming a multiplicative model of genetic interactions. Green circles, observed growth of double siRNA-treated cells. For B, viability was determined by high-content imaging of nuclei 4 d after transfection. For C, viability was determined by a colony formation assay 10 d after transfection.

Our data predict that human cancer cells with certain CIN mutations should be sensitized to perturbation of the aforementioned replisome components. Thus, we attempted to recapitulate some of the genetic interactions in human cells by using siRNA-mediated knockdown of the human orthologs of selected members of the network shown in Figure 4A. We used the karyotypically stable, near-diploid colorectal cancer cell line HCT116 as a model system (McLellan *et al.* 2012; McManus *et al.* 2009).We targeted MCM2, MCM10, GINS1/PSF1, CDC45L, and POLA1 for knockdown by siRNA and asked whether these treatments sensitized cells to chemical inhibition of MRE11A with mirin, a recently described inhibitor of Mre11-Rad50-Nbs1 complex activity (Dupre *et al.* 2008). We found that knockdown of MCM10 did not sensitize cells to mirin treatment, whereas knockdown of the other four targets significantly reduced cell viability in the presence of mirin (Figure 5A). We validated this chemical-genetic interaction by using dual siRNA-mediated knockdown, depleting cells of MRE11A simultaneously with MCM2, PSF1, CDC45L, or POLA1. We found that, 4 d after transfection, the number of cells remaining after dual knockdown treatments was less than the predicted product of that of the two single knockdowns, indicative of a synergistic effect of the two siRNAs (Figure 5B). These genetic interactions were confirmed by a colony formation assay (Figure 5C). Although we acknowledge that our results only reflect the genetic interaction network in a single cell line, taken together, these data support the concept of using biological network information to predict alternative therapeutic genetic interactions when a promising target (*i.e.*, Ctf4) is not readily druggable.

## Discussion

Defining SL genetic interactions is a promising avenue for rational prediction of therapeutic targets for cancer because tumors are genetically distinct from surrounding tissue. In this work, we develop approaches to identify candidate therapeutic genetic interactions through focused or genome-wide screens in yeast centered on either cancer-gene orthologs or prospective therapeutic targets. One important outcome of this work is validation of the supposition that genes required for chromosome stability will have negative genetic interactions with other genes required for chromosome stability. This simple observation is remarkably important to how we currently treat many cancers (*i.e.*, with DNA damaging chemicals, radiation or mitotic spindle inhibitors).

Our analysis of SL partners of CIN cancer genes implicates genes involved in DNA replication and repair as widely connected candidate therapeutic targets, along with some novel targets such as P-bodies. We anticipate this network evolving considerably as more recurrently somatically mutated CIN cancer genes are discovered by large-scale tumor-genome sequencing efforts. We also note that essential genes are currently underrepresented in the SL network of data that is publicly available for CIN genes because most high-throughput genetic interaction screening to date has focused on nonessential yeast genes. Nearly half of all CIN genes are essential, and the inclusion of a large essential gene SL network should dramatically enrich this data set and the number of potential targets. We favor a model where selective killing by some current therapies is specifically linked to the CIN mutant genetic background of the tumor and not only to the tumor-associated hyperproliferative phenotype. Selective killing by drastically increasing the mutation and/or aneuploidy rates is an accepted mechanism and is consistent with the improved prognosis associated with very high levels of CIN in some tumors (Birkbak *et al.* 2011). The conditions that most specifically aggravate CIN/hyper-mutability toward lethality will depend on the genetic background of the tumor. This highlights the importance of combining somatic mutation detection by deep-sequencing with functional studies of CIN phenotypes in tumors to understand and improve current therapies.

The eukaryotic DNA replication fork contains dozens of proteins, and although the complete genetic interaction space of the fork is not known because most of the components are essential, many of these could be therapeutic targets based on our analysis. We successfully predicted genetic interactions of Ctf4-interacting proteins at the replication fork with cancer gene orthologs. The yeast DNA replication fork mutants we tested exhibited increased cell cycle arrest and recombination centers, indicating excess DNA damage or inefficient repair. One model is that defects in replication lead to increased replisome stalling that requires functional Mre11 and Sgs1 for fork restart or DNA repair (such as by template switching or homologous recombination) (Torres *et al.* 2004). Unrepaired or not-yet-replicated DNA triggers a G2/M arrest, and it is known that DNA damage can signal to the spindle assembly checkpoint, which acts subsequently to reinforce the arrest independent of kinetochore function (Kim and Burke 2008). Therefore, the common DNA damage phenotypes of the replisome mutants described here could explain the SL relationship with cancer CIN genes involved in DNA repair (*MRE11*/*SGS1*) and the cell cycle (*BUB1*). These predicted genetic interactions are conserved from yeast to humans, suggesting novel avenues for therapeutic development. It is probable that these Ctf4-surrogate interactors may ultimately prove to be superior drug targets. In particular, the MCM helicase complex and POLA1 each possess enzymatic activity, and thus may be more amenable to *in vitro* biochemical assay development and large-scale screens for small-molecule inhibitors. Overall, this invokes two important concepts: first, that the functional partners of candidate therapeutic hub genes may represent additional targets based on shared genetic interactions, and second, that the DNA replication fork is a hub of SL interactions with CIN cancer genes.

The concept of targeting numerous DNA replication fork components for therapeutic development is exciting given the early success of PARP inhibitors for SL targeting of cancers (reviewed in Brough *et al.* 2011). PARP mediates replication fork stability in response to stress and we recently showed that depletion of the cohesin SMC1A, which is mutated in colorectal cancer, sensitizes cells to selective killing by PARP inhibition (McLellan *et al.* 2012). Other studies have shown selective killing by PARP inhibition of cells from diverse tumor types bearing mutations in *ATM*, *MRE11A*, *BRCA2*, or the *EWS-FLI1* translocation, suggesting that PARP is a *bona fide* hub for genetic interactions with cancer genes (Brenner *et al.* 2012; Bryant *et al.* 2005; Farmer *et al.* 2005; Vilar *et al.* 2011; Williamson *et al.* 2010). Although several DNA repair proteins have gained traction as tumor-selective SL therapeutic targets, our data and the literature suggest that DNA replication fork proteins themselves are also potentially hub therapeutic targets and could serve as a broad-spectrum means to selectively kill cancer cells.

## Supplementary Material

Supporting Information
