# Supplementary material for: Saccharomyces cerevisiae Genetics Predicts Candidate Therapeutic Genetic Interactions at the Mammalian Replication Fork
Source: G3 (Bethesda). 2013 Feb 1;3(2):273–82. doi: 10.1534/g3.112.004754 (PMC3564987; doi:10.1534/g3.112.004754)
Supplement: Supporting Information [file supp_3_2_273__index.html]

Supporting Information 

# *Saccharomyces cerevisiae* Genetics Predicts Candidate Therapeutic Genetic Interactions at the Mammalian Replication Fork

## Supporting Information for van Pel *et al.*, 2013

**Files in this Data Supplement:**

- File S1 - Figures S1-S4 and Tables S1-S8 (PDF, 1 MB)
- Figure S1 - *TAF1/TAF1L* mutations and genetic interaction partners (PDF, 215 KB)
- Figure S2 - Ctf4 is physically and functionally linked to several replication protein complexes. (PDF, 383 KB)
- Figure S3 - Expanding the therapeutic value of *CTF4* (PDF, 102 KB)
- Figure S4 - Human *CTF4/WDHD1* overexpression is non-toxic to yeast. (PDF, 1 MB)
- Table S6 - Quantitation of colony sectoring and chromatid separation assay for *CTF4* mutants (PDF, 66 KB)
- Table S1 - Yeast strains used in this study (.xls, 35 KB)
- Table S2 - Chemical-genetic interaction screening raw data (.xls, 1 MB)
- Table S3 - Compiled chemical sensitivity data from SGD and this study (.xls, 136 KB)
- Table S4 - SGA raw scores for essential miniarray screens and *taf1-1* whole genome screen (.xls, 3 MB)
- Table S5 - Summary of tetrad analysis for all SGA screens (.xls, 29 KB)
- Table S7 - Compiled *CTF4* genetic interactors and their mutational status in cancer (.xls, 121 KB)
- Table S8 - SGA raw scores *ctf4*Δ essential miniarray screen (.xls, 289 KB)
