# Supplementary material for: Saccharomyces cerevisiae Genetics Predicts Candidate Therapeutic Genetic Interactions at the Mammalian Replication Fork
Source: G3 (Bethesda). 2013 Feb 1;3(2):273–82. doi: 10.1534/g3.112.004754 (PMC3564987; doi:10.1534/g3.112.004754)
Supplement: Supporting Information [file supp_3.2.273_004754SI.pdf]

G3 Genes | Genomes | Genetics

Saccharomyces cerevisiae genetics predicts candidate therapeutic genetic interactions at the mammalian replication fork

Derek M. van Pel*, Peter C. Stirling*, Sean W. Minaker, Payal Sipahimalani and Philip Hieter.

Michael Smith Laboratories, 2185 East Mall University of British Columbia, Vancouver, British Columbia, Canada, V6T1Z4

* Authors contributed equally.

Correspondence to P. Hieter. Email: Hieter@msl.ubc.ca

Telephone: 1-604-822-5115

Fax: 1-604-822-2114

DOI: 10.1534/g3.112.004754

File S1

Functional analysis of Ctf4 mutant alleles

Two proteins of disparate functions that bind Ctf4 are the GINS complex member and DNA replication protein Sld5, and Mms22, a subunit of an E3-ubiquitin ligase required for double strand break repair (Ben-Aroya *et al*. 2010; Duro *et al*. 2010; O'Donnell *et al*. 2010). We performed a yeast two-hybrid assay assessing the ability of the nine Ctf4 alleles to bind Sld5 and Mms22. As in published reports, the interaction between Ctf4 and Sld5 is sensitive to perturbation by mutation at all points tested except the extreme N-terminus (Gambus *et al*. 2009); on the other hand, the interaction between Ctf4 and Mms22 appears to be most sensitive to mutation or deletion in the middle third of the Ctf4 primary sequence (**Figure S2**).

Knowing that there were differences in protein interactions between the Ctf4 alleles, we wanted to correlate phenotypic severity with the differing physical interactions. We first compared the sensitivity of the allele series to bleomycin, a radiomimetic that induces DNA double strand breaks, and HU (**Figure S2**). Alleles that retained interactions with both Sld5 and Mms22 were not sensitive to genotoxic stress (i.e. ctf4-107 and ctf4-41). Conversely, alleles with disrupted Sld5 binding had a range of sensitivities from very subtle phenotypes in ctf4-154, to intermediate phenotypes in ctf4-46, -43 and -66 to null phenotypes in ctf4-25, -50 and -65 (**Figure S2C**). Binding to Mms22 did not predict a consistent trend in drug sensitivity. This experiment is complicated by the fact that the status of the many other Ctf4 physical interactions is unknown and the expression levels and protein stability of the various alleles are unknown. We quantitated the genome stability defects in each *CTF4* allele first using a quantitative Chromosome Transmission Fidelity (CTF) assay (Spencer *et al*. 1990). While all alleles tested had a strong increase in chromosome loss, there were no significant differences between the alleles in this assay (**Table S6**). The CTF method represents a sensitized assay in which the chromosome fragment is prone to loss and therefore may have prevented our identification of subtle differences between the alleles. We also performed a sister chromatid cohesion assay (Michaelis *et al*. 1997). Again, all but one of the alleles had an elevated frequency of cells with separated chromatids similar to or greater than *ctf4*Δ (**Table S6**). The presence of cohesion defects greater than *ctf4*Δ hints that some of the alleles may have dominant negative phenotypic effects. *ctf4-41* exhibited lower rates of cohesion loss than the other mutants (**Table S6**). Interestingly, ctf4-41 was the only mutant protein that was able to physically interact with both Sld5 and Mms22 (**Figure S2**). Taken together, these data suggest that the ability to bind efficiently to Sld5 is critical to the cohesion establishment and genotoxin resistance functions Ctf4. Furthermore, the ability of a Ctf4 mutant protein to interact with Mms22 is predictive of neither its cohesion establishing ability, nor of its ability to function in the response to DNA damage.

Expanding the therapeutic target range of Ctf4/WDHD1 inhibitors

CTF4 is a genetic interaction hub connected to the yeast orthologs of CIN cancer genes (Yuen *et al*. 2007). Thus, Ctf4/WDHD1 represents a potential broad-spectrum target for anticancer therapeutic development. As genetic interaction data is not yet available for a large proportion of the essential yeast genome, we sought to expand the range of genotypes targetable with potential inhibitors of Ctf4/WDHD1 by carrying out an SGA screen of Ctf4 against collections of essential gene mutants (Breslow *et al*. 2008; Li *et al*. 2011). In addition to yielding candidate gene-drug target interactions, this approach also defines the potential off-target effects of Ctf4 inhibition by revealing the complement of cellular pathways sensitized to Ctf4 perturbation.

Figure S3 shows the network of essential genes sensitized to *CTF4* deletion. A handful of interactions were validated by tetrad analysis and spot-dilution assays (**Table S5**). GO term analysis showed that the synthetic lethal partner set is enriched for genes involved in DNA replication, response to DNA damage, cell cycle progression, and mitotic spindle dynamics, all of which are consistent with previously described roles for Ctf4. Combining our data with previously published CTF4 genetic interactions for nonessential genes, we identified synthetic lethal interactions with numerous mutations that are recurrent in tumors (**Table S7**). For example, *ctf4Δ* interacts negatively with mutations in the essential genes *SMC1/MCD1*, *SMC3*, *CDC4*, *SCC2, SCC1/IRR1,* and the nonessential gene *MRE11* (this study and (McLellan *et al*. 2009)), the human orthologs of which account for >15% of the mutational spectrum in colorectal cancer (Barber *et al*. 2008; Rajagopalan *et al*. 2004; Wang *et al*. 2004). Thus, *CTF4* genetic interaction partners can be recurrently mutated in tumors, suggesting that a personalized, tumor genome sequencing-based approach to treatment could indicate whether a hypothetical Ctf4/WDHD1 inhibitor would be effective. We note that a mutation in a human ortholog of a *CTF4* SL partner need not be causal in tumorigenesis to sensitize tumor cells to inhibition of Ctf4/WDHD1 function.

The SL interactions of cancer-gene orthologs with *ctf4*Δ presumably reflect a key deficiency in the replisome or DNA repair that is linked to the *CTF4* deletion phenotype. Indeed, in another recent study we have confirmed by siRNA that depletion of *CTF4/WDHD1* in human cancer cells sensitizes them to depletion of known cancer genes *MRE11A*, *CDC4*, cohesins or the Bloom's syndrome helicase, strongly supporting Ctf4 as a therapeutic target (van Pel et al., 2012).

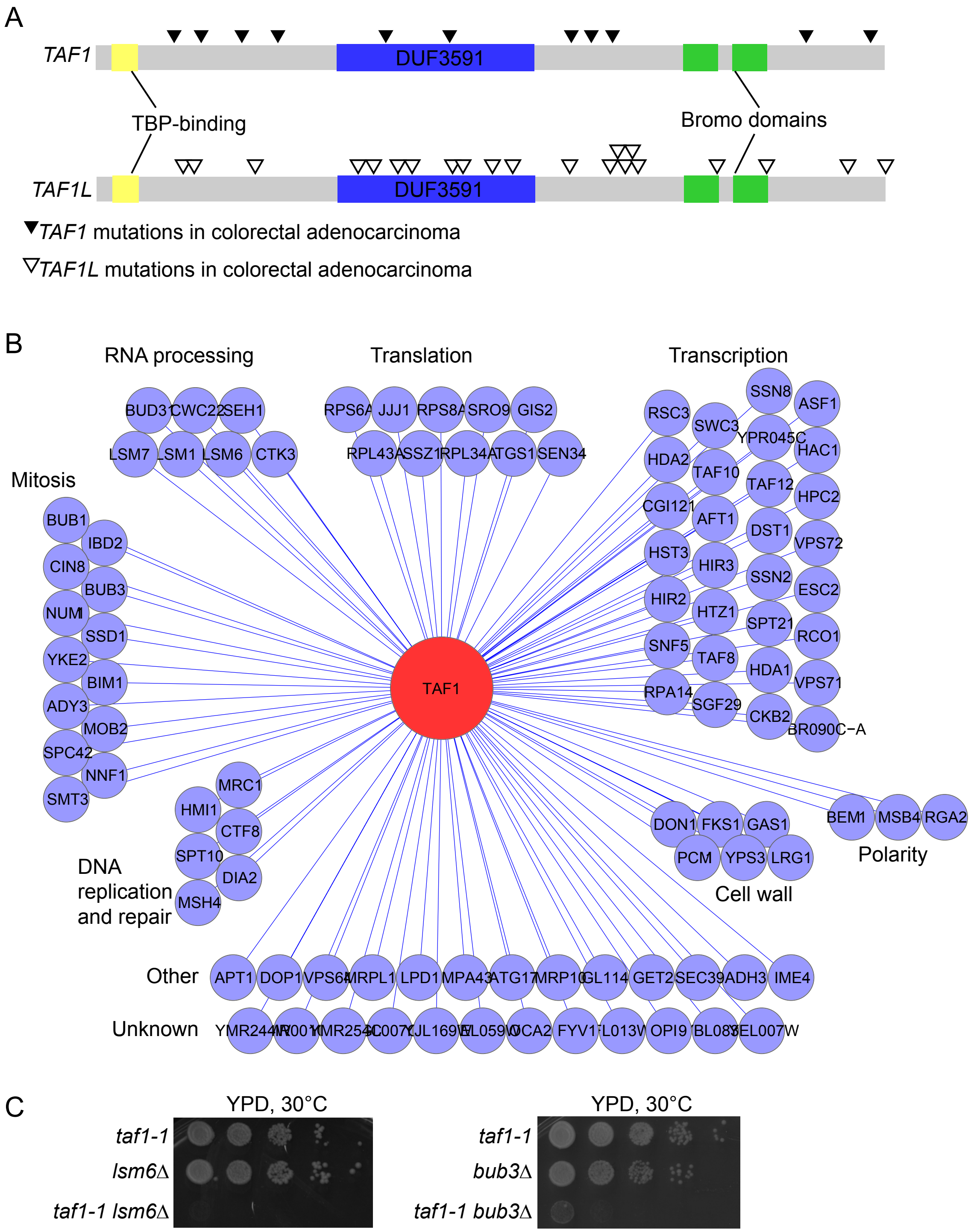

Figure S1 *TAF1/TAF1L* mutations and genetic interaction partners. (A) Schematic of human *TAF1* orthologs showing missense mutations in a set of colorectal adenocarcinoma (TCGA data via MSKCC www.cbioportal.org/public_portal). Mutations are indicated at their relative position with triangles. (B) Network of negative genetic interactions identified with the yeast *taf1-1* allele. (C) Tetrad analysis and spot dilution assays to validate selected interactions from B are summarized in **Table S5**. Shown are interactions with genes representing hub processes hit by many CIN mutations.

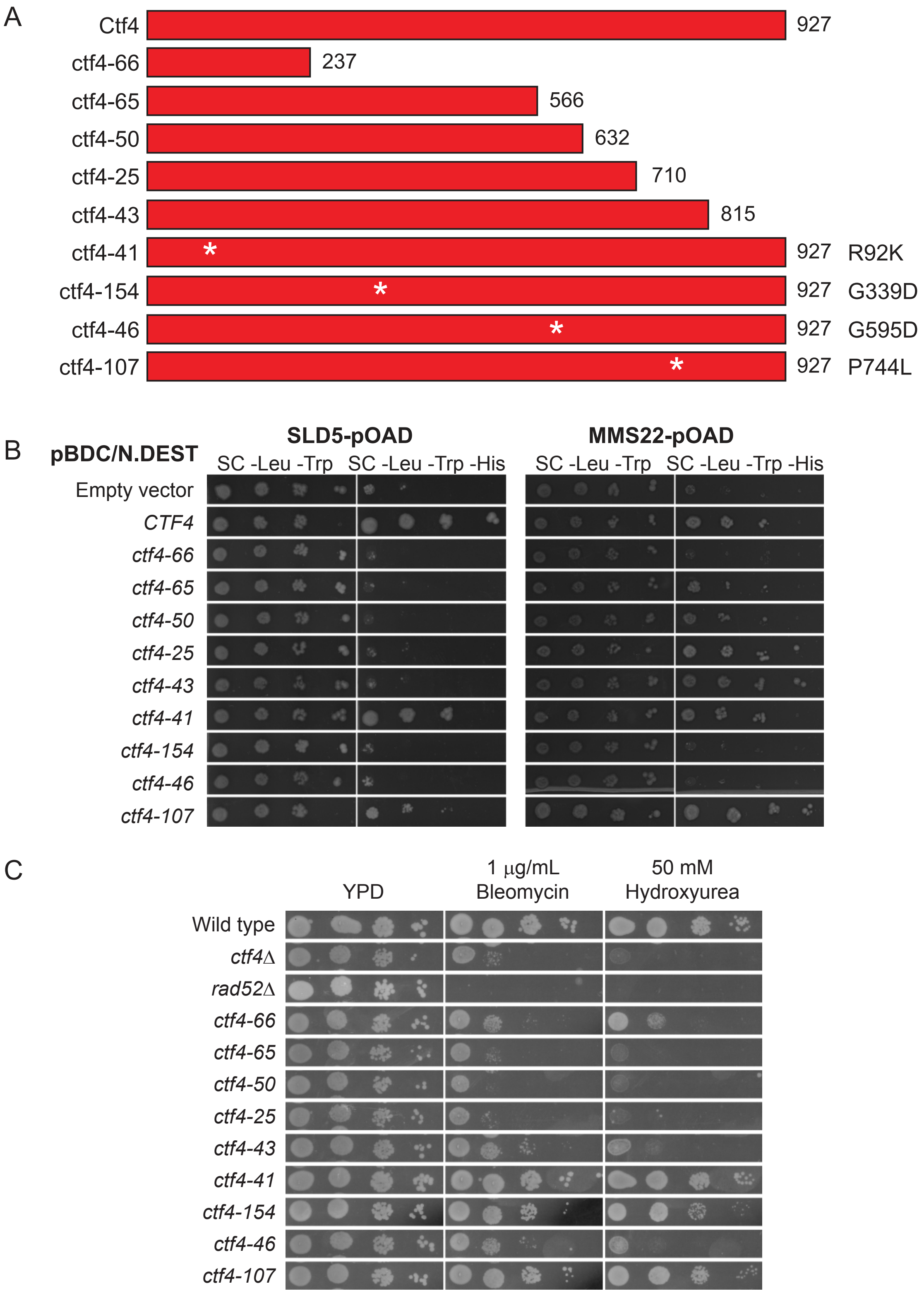

Figure S2 Ctf4 is physically and functionally linked to several replication protein complexes. (A) Schematic of *CTF4* alleles used. Numbers represent the amino acid number and asterisks indicate the relative position of point mutations. (B) *CTF4* alleles confer differential ability to interact with Sld5 and Mms22 by yeast-two-hybrid. Cells carrying the indicated plasmids were grown to log phase, subjected to ten-fold serial dilution, plated on the indicated medium, and imaged after five days' growth. (C) *CTF4* alleles confer differential sensitivity to DNA damaging drugs. Experiment was conducted as in (B) on plates containing the indicated drug and concentration.

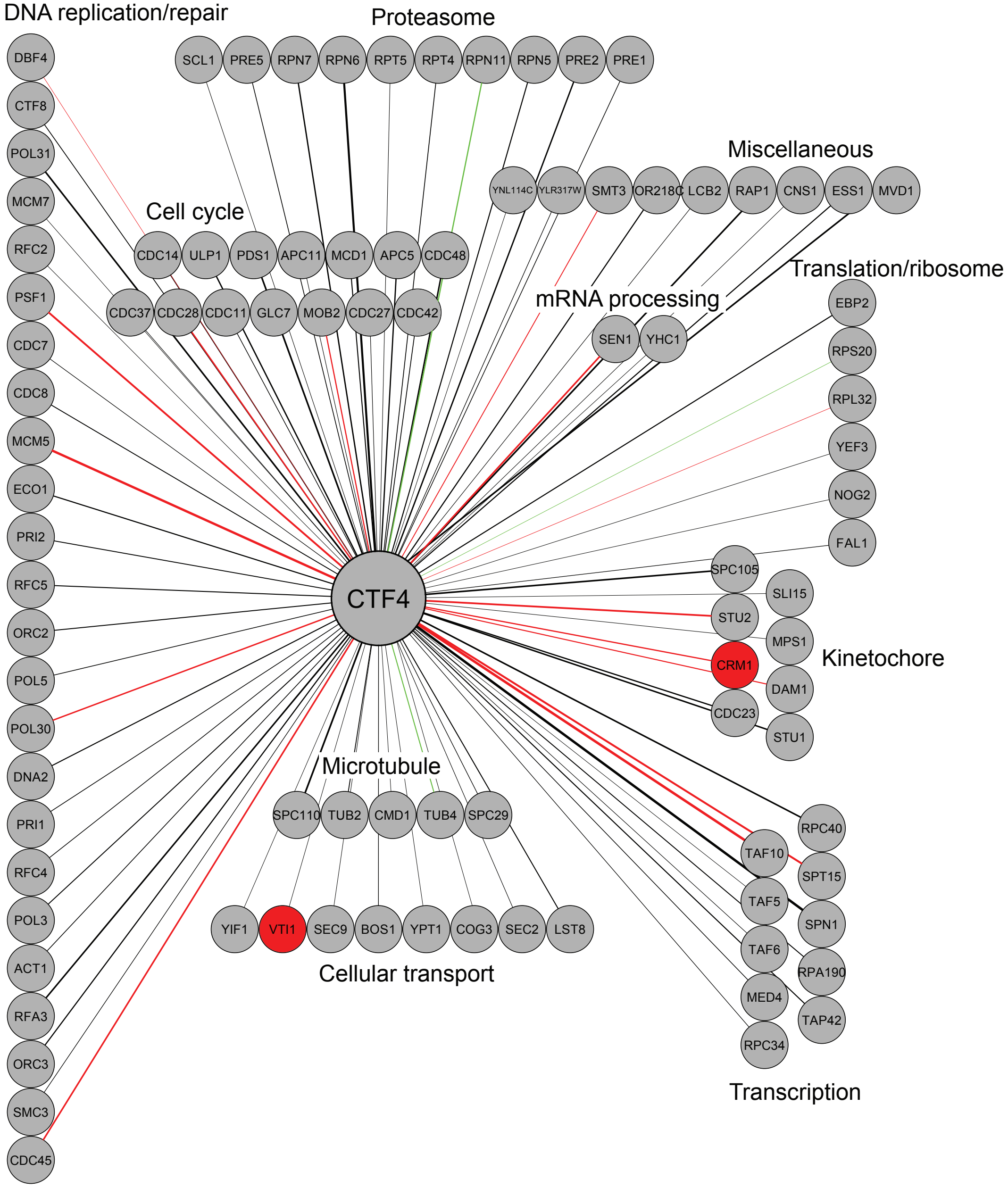

Figure S3 Expanding the therapeutic value of *CTF4*. (A) Genetic interactions between *CTF4* and essential genes determined by SGA analysis. Edge thickness represents relative strength of interaction. Red edges, interaction validated by tetrad or spot dilution analysis (**Table S5**). Green edge, interaction did not validate by tetrad or spot dilution analysis. Red node, human ortholog appears in the cancer gene census.

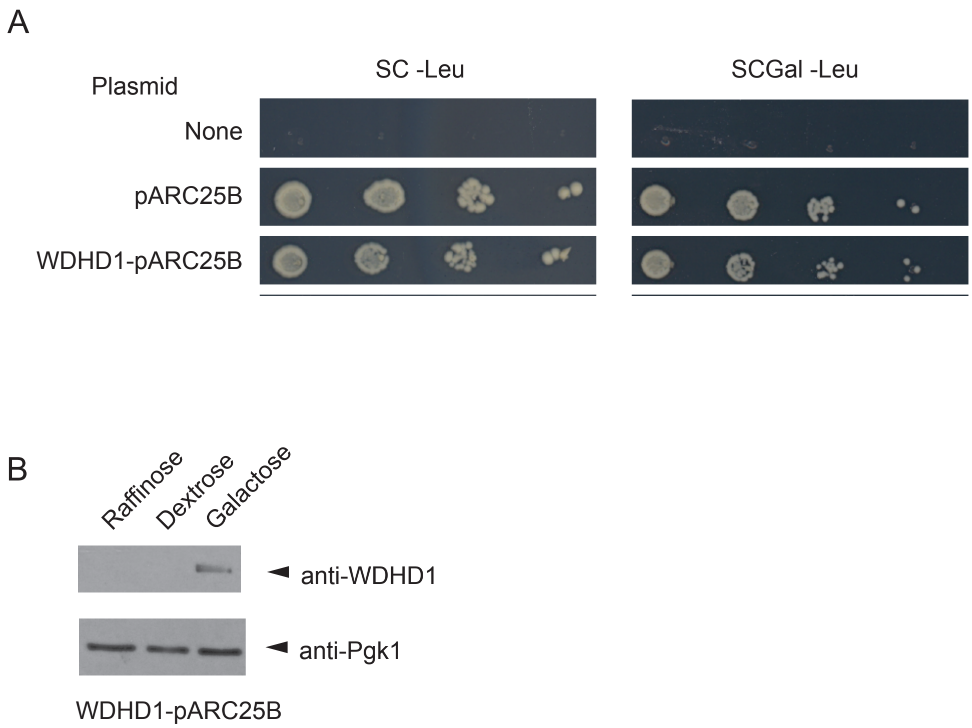

Figure S4 Human *CTF4/WDHD1* overexpression is non-toxic to yeast. (A) Yeast expressing *WDHD1* from a 2m galactose-driven overexpression plasmid (pARC25B), were spot diluted onto selective media with dextrose or galactose. Growth was comparable between *WDHD1*-overexpressing plasmid and an empty vector. (B) *WDHD1* is expressed from pARC25B. Western analysis of yeast lysates show that WDHD1 is expressed under the conditions used in A.

Tables S1-S8

Available for download as Excel files at http://www.g3journal.org/lookup/suppl/doi:10.1534/g3.112.004754/-/DC1.

Table S1 Yeast strains used in this study.
Table S2 Chemical-genetic interaction screening raw data.
Table S3 Compiled chemical sensitivity data from SGD and this study.
Table S4 SGA raw scores for essential miniarray screens and *taf1-1* whole genome screen.
Table S5 Summary of tetrad analysis for all SGA screens.
Table S6 Quantitation of colony sectoring and chromatid separation assay for *CTF4* mutants (on next page).
Table S7 Compiled *CTF4* genetic interactors and their mutational status in cancer.
Table S8 SGA raw scores *ctf4*Δ essential miniarray screen.

Table S6 Quantitation of colony sectoring and chromatid separation assay for CTF4 mutants.

Allele	Half-sectored colonies (%, n)	Fold increase in half-sectored colonies over wild type	α factor-arrested cells with separated chromatids (%)	Nocodazole-arrested cells with separated chromatids (% ± SEM)
WT	0.031, 3185	N/A	2.0	4.1 ± 1.2
ctf4Δ	1.72, 1981	55	2.5	21.9 ± 3.5
ctf4-66	1.40, 1002	45	2.0	35.8 ± 0.8
ctf4-65	1.94, 2111	23	8.0	24.4 ± 3.4
ctf4-50	0.78, 385	25	7.0	33.2 ± 0.4
ctf4-25	1.94, 2111	62	3.2	27.3 ± 4.5
ctf4-43	1.79, 4964	57	3.8	26.7 ± 2.5
ctf4-41	ND	N/A	1.0	13.4 ± 1.6
ctf4-154	0.92, 1518	29	2.0	27.0 ± 4.0
ctf4-46	1.26, 1661	40	3.0	21.6 ± 0.3
ctf4-107	1.23, 1873	39	0.0	29.2 ± 3.6

Supporting Methods

Genome stability assays

The Chromosome Transmission Fidelity assay was performed as described (Spencer *et al*. 1990). The sister chromatid cohesion assay was performed as described (Ben-Aroya *et al*. 2008).

Yeast two-hybrid

Yeast two-hybrid assay was performed by mating strains carrying plasmids expressing proteins of interest fused to the GAL4 activating domain or DNA binding domain. Cells were grown to log phase, then cultures were subjected to serial ten-fold dilutions and plated on SC –Leu –Trp and SC –Leu –Trp –His. pOAD encodes the GAL4 activating domain; pOBD2 encodes the GAL4 DNA-binding domain; pBDC/N.DEST is a Gateway-compatible vector designed in this study that encodes the GAL4 DNA-binding domain, and is a derivative of pBDC, itself a derivative of pOBD2.

LITERATURE CITED

Barber, T. D., K. McManus, K. W. Yuen, M. Reis, G. Parmigiani *et al*, 2008 Chromatid cohesion defects may underlie chromosome instability in human colorectal cancers. Proc. Natl. Acad. Sci. U. S. A. **105:** 3443-3448.

Ben-Aroya, S., C. Coombes, T. Kwok, K. A. O'Donnell, J. D. Boeke *et al*, 2008 Toward a comprehensive temperature-sensitive mutant repository of the essential genes of saccharomyces cerevisiae. Mol. Cell **30:** 248-258.

Ben-Aroya, S., N. Agmon, K. Yuen, T. Kwok, K. McManus *et al*, 2010 Proteasome nuclear activity affects chromosome stability by controlling the turnover of Mms22, a protein important for DNA repair. PLoS Genet. **6:** e1000852.

Breslow, D. K., D. M. Cameron, S. R. Collins, M. Schuldiner, J. Stewart-Ornstein *et al*, 2008 A comprehensive strategy enabling high-resolution functional analysis of the yeast genome. Nat. Methods **5:** 711-718.

Duro, E., C. Lundin, K. Ask, L. Sanchez-Pulido, T. J. MacArtney *et al*, 2010 Identification of the MMS22L-TONSL complex that promotes homologous recombination. Mol. Cell **40:** 632-644.

Gambus, A., F. van Deursen, D. Polychronopoulos, M. Foltman, R. C. Jones *et al*, 2009 A key role for Ctf4 in coupling the MCM2-7 helicase to DNA polymerase alpha within the eukaryotic replisome. EMBO J. **28:** 2992-3004.

Li, Z., F. J. Vizeacoumar, S. Bahr, J. Li, J. Warringer *et al*, 2011 Systematic exploration of essential yeast gene function with temperature-sensitive mutants. Nat. Biotechnol. **29:** 361-367.

McLellan, J., N. O'Neil, S. Tarailo, J. Stoepel, J. Bryan *et al*, 2009 Synthetic lethal genetic interactions that decrease somatic cell proliferation in caenorhabditis elegans identify the alternative RFC CTF18 as a candidate cancer drug target. Mol. Biol. Cell **20:** 5306-5313.

Michaelis, C., R. Ciosk and K. Nasmyth, 1997 Cohesins: Chromosomal proteins that prevent premature separation of sister chromatids. Cell **91:** 35-45.

O'Donnell, L., S. Panier, J. Wildenhain, J. M. Tkach, A. Al-Hakim *et al*, 2010 The MMS22L-TONSL complex mediates recovery from replication stress and homologous recombination. Mol. Cell **40:** 619-631.

Rajagopalan, H., P. V. Jallepalli, C. Rago, V. E. Velculescu, K. W. Kinzler *et al*, 2004 Inactivation of hCDC4 can cause chromosomal instability. Nature **428:** 77-81.

Spencer, F., S. L. Gerring, C. Connelly and P. Hieter, 1990 Mitotic chromosome transmission fidelity mutants in saccharomyces cerevisiae. Genetics **124:** 237-249.

Van Pel, D.M., I.J. Barrett, Y. Shimizu, B.V. Sajesh, B.J. Guppy *et al*, 2012 An Evolutionarily Conserved Synthetic Lethal Interaction Network Identifies FEN1 as a Broad-spectrum Target for Anticancer Therapeutic Development. PLoS Genet. **In Press**.

Wang, Z., J. M. Cummins, D. Shen, D. P. Cahill, P. V. Jallepalli *et al*, 2004 Three classes of genes mutated in colorectal cancers with chromosomal instability. Cancer Res. **64:** 2998-3001.

Yuen, K. W., C. D. Warren, O. Chen, T. Kwok, P. Hieter *et al*, 2007 Systematic genome instability screens in yeast and their potential relevance to cancer. Proc. Natl. Acad. Sci. U. S. A. **104:** 3925-3930.
