# Supplementary material for: Saccharomyces cerevisiae Genetics Predicts Candidate Therapeutic Genetic Interactions at the Mammalian Replication Fork
Source: G3 (Bethesda). 2013 Feb 1;3(2):273–82. doi: 10.1534/g3.112.004754 (PMC3564987; doi:10.1534/g3.112.004754)
Supplement: Supporting Information [file supp_3.2.273_FigureS1.pdf]

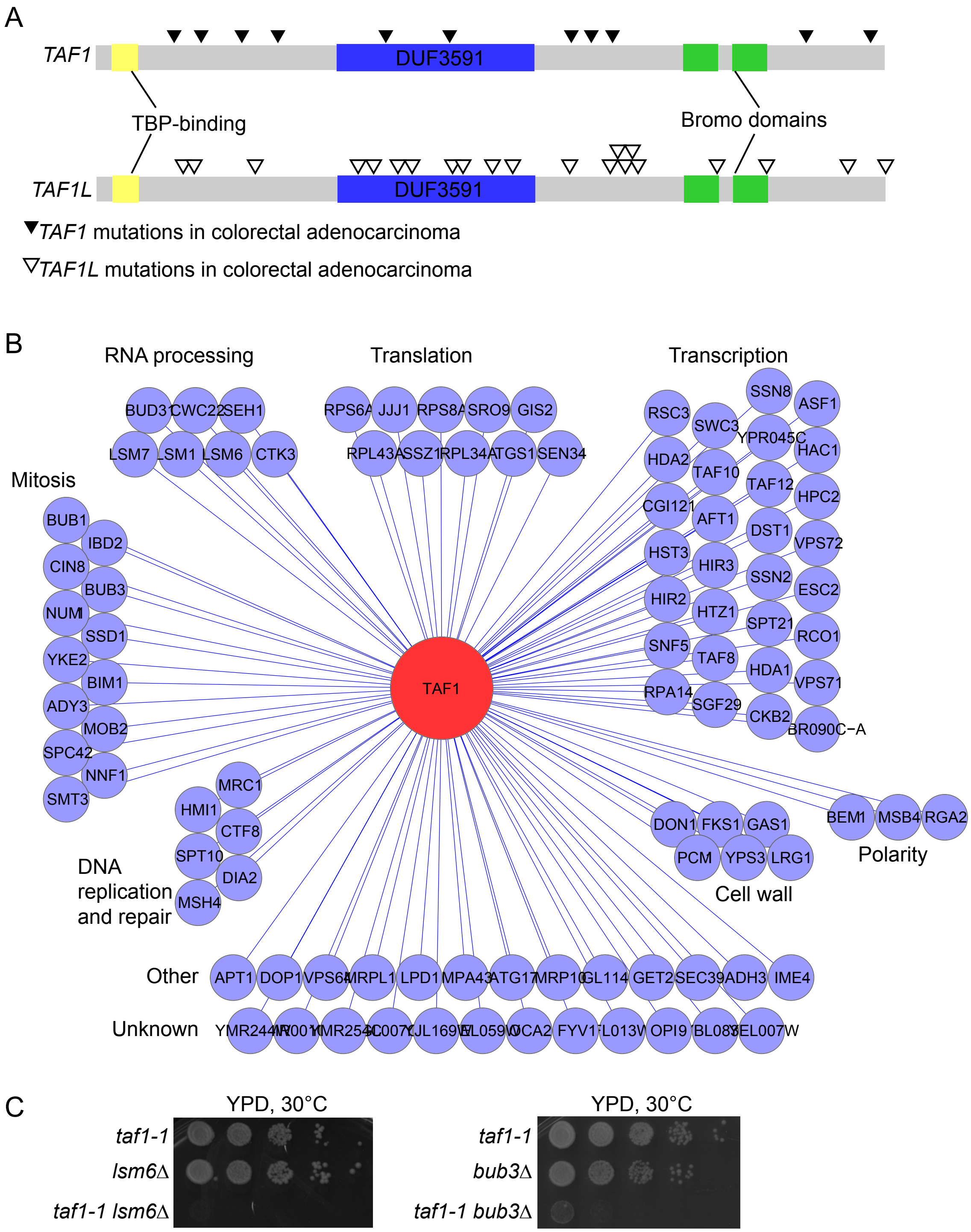

Figure S1 *TAF1/TAF1L* mutations and genetic interaction partners. (A) Schematic of human *TAF1* orthologs showing missense mutations in a set of colorectal adenocarcinoma (TCGA data via MSKCC www.cbioportal.org/public_portal). Mutations are indicated at their relative position with triangles. (B) Network of negative genetic interactions identified with the yeast *taf1-1* allele. (C) Tetrad analysis and spot dilution assays to validate selected interactions from B are summarized in **Table S5**. Shown are interactions with genes representing hub processes hit by many CIN mutations.
