# Supplementary material for: Saccharomyces cerevisiae Genetics Predicts Candidate Therapeutic Genetic Interactions at the Mammalian Replication Fork
Source: G3 (Bethesda). 2013 Feb 1;3(2):273–82. doi: 10.1534/g3.112.004754 (PMC3564987; doi:10.1534/g3.112.004754)
Supplement: Supporting Information [file supp_3.2.273_FigureS2.pdf]

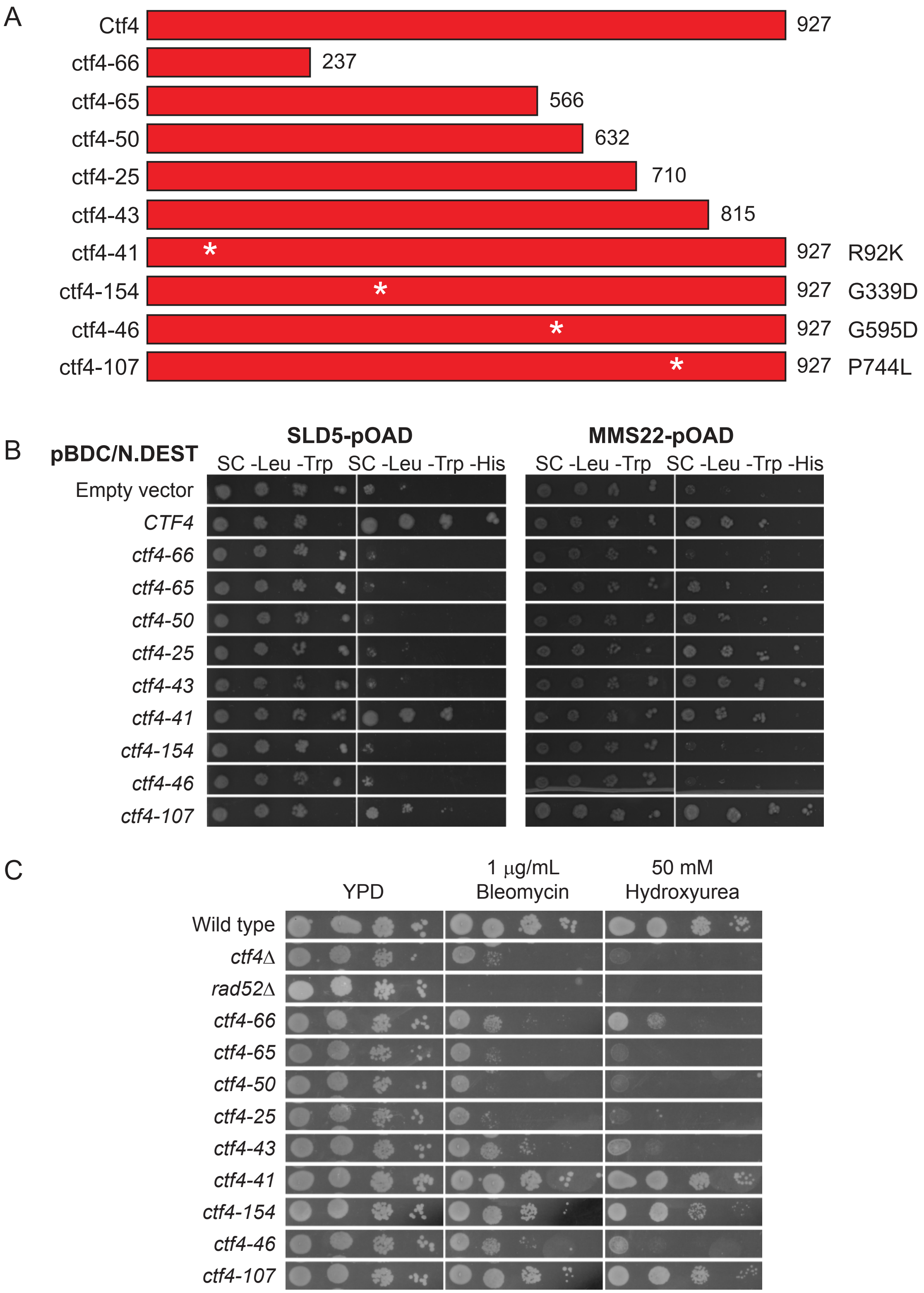

Figure S2 Ctf4 is physically and functionally linked to several replication protein complexes. (A) Schematic of *CTF4* alleles used. Numbers represent the amino acid number and asterisks indicate the relative position of point mutations. (B) *CTF4* alleles confer differential ability to interact with Sld5 and Mms22 by yeast-two-hybrid. Cells carrying the indicated plasmids were grown to log phase, subjected to ten-fold serial dilution, plated on the indicated medium, and imaged after five days' growth. (C) *CTF4* alleles confer differential sensitivity to DNA damaging drugs. Experiment was conducted as in (B) on plates containing the indicated drug and concentration.
