# Supplementary material for: Saccharomyces cerevisiae Genetics Predicts Candidate Therapeutic Genetic Interactions at the Mammalian Replication Fork
Source: G3 (Bethesda). 2013 Feb 1;3(2):273–82. doi: 10.1534/g3.112.004754 (PMC3564987; doi:10.1534/g3.112.004754)
Supplement: Supporting Information [file supp_3.2.273_FigureS3.pdf]

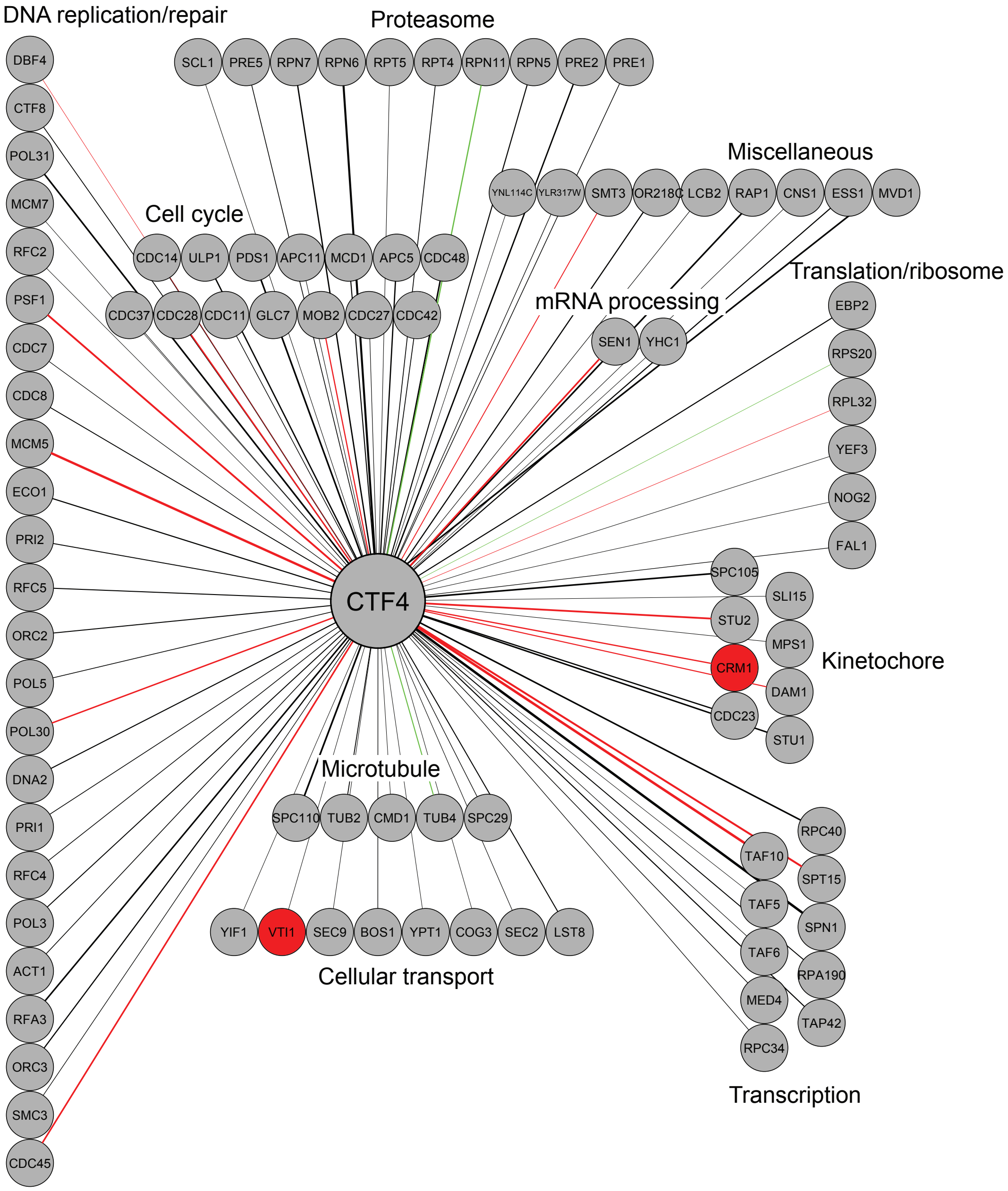

Figure S3 Expanding the therapeutic value of *CTF4*. (A) Genetic interactions between *CTF4* and essential genes determined by SGA analysis. Edge thickness represents relative strength of interaction. Red edges, interaction validated by tetrad or spot dilution analysis (**Table S5**). Green edge, interaction did not validate by tetrad or spot dilution analysis. Red node, human ortholog appears in the cancer gene census.
