# Supplementary material for: Saccharomyces cerevisiae Genetics Predicts Candidate Therapeutic Genetic Interactions at the Mammalian Replication Fork
Source: G3 (Bethesda). 2013 Feb 1;3(2):273–82. doi: 10.1534/g3.112.004754 (PMC3564987; doi:10.1534/g3.112.004754)
Supplement: Supporting Information [file supp_3.2.273_FigureS4.pdf]

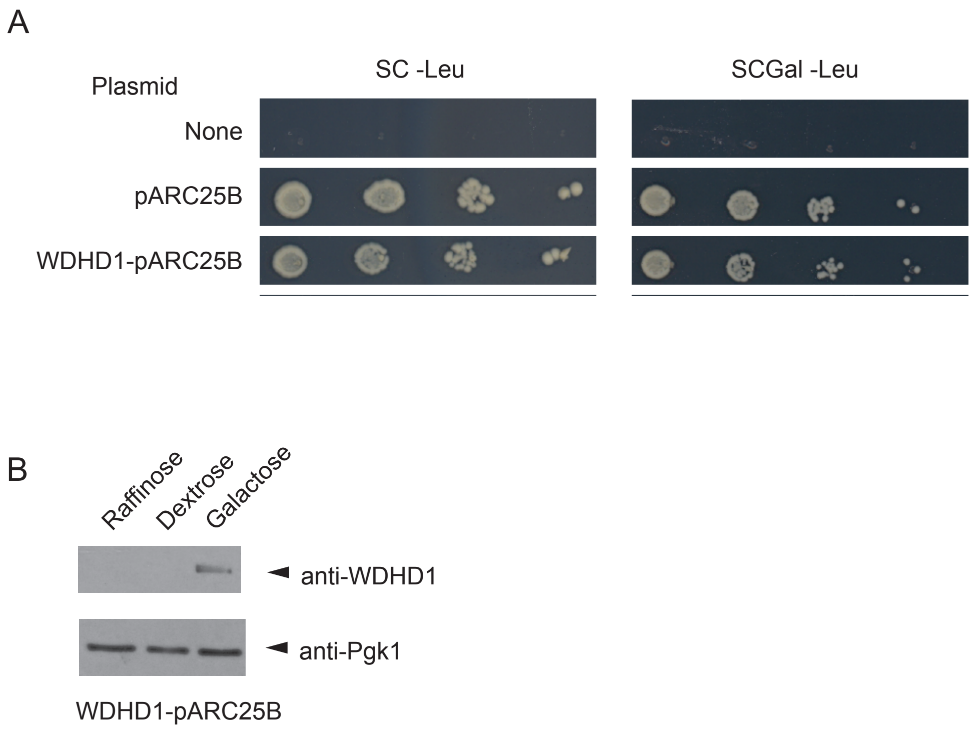

Figure S4 Human *CTF4/WDHD1* overexpression is non-toxic to yeast. (A) Yeast expressing *WDHD1* from a 2m galactose-driven overexpression plasmid (pARC25B), were spot diluted onto selective media with dextrose or galactose. Growth was comparable between *WDHD1*-overexpressing plasmid and an empty vector. (B) *WDHD1* is expressed from pARC25B. Western analysis of yeast lysates show that WDHD1 is expressed under the conditions used in A.
