# Supplementary material for: Saccharomyces cerevisiae Genetics Predicts Candidate Therapeutic Genetic Interactions at the Mammalian Replication Fork
Source: G3 (Bethesda). 2013 Feb 1;3(2):273–82. doi: 10.1534/g3.112.004754 (PMC3564987; doi:10.1534/g3.112.004754)
Supplement: Supporting Information [file supp_3.2.273_TableS6.pdf]

Table S6 Quantitation of colony sectoring and chromatid separation assay for CTF4 mutants.

Allele	Half-sectored colonies (%, n)	Fold increase in half-sectored colonies over wild type	α factor-arrested cells with separated chromatids (%)	Nocodazole-arrested cells with separated chromatids (% ± SEM)
WT	0.031, 3185	N/A	2.0	4.1 ± 1.2
ctf4Δ	1.72, 1981	55	2.5	21.9 ± 3.5
ctf4-66	1.40, 1002	45	2.0	35.8 ± 0.8
ctf4-65	1.94, 2111	23	8.0	24.4 ± 3.4
ctf4-50	0.78, 385	25	7.0	33.2 ± 0.4
ctf4-25	1.94, 2111	62	3.2	27.3 ± 4.5
ctf4-43	1.79, 4964	57	3.8	26.7 ± 2.5
ctf4-41	ND	N/A	1.0	13.4 ± 1.6
ctf4-154	0.92, 1518	29	2.0	27.0 ± 4.0
ctf4-46	1.26, 1661	40	3.0	21.6 ± 0.3
ctf4-107	1.23, 1873	39	0.0	29.2 ± 3.6
